# Health-Related Quality of Life and Injuries in Physical Education Students: A Multi-Group Model According to the Degree of Adherence to the Mediterranean Diet

**DOI:** 10.3390/ejihpe14050075

**Published:** 2024-04-24

**Authors:** Eduardo Melguizo-Ibáñez, José Luis Ubago-Jiménez, Daniel Sanz-Martín, José Manuel Alonso-Vargas

**Affiliations:** 1 Department of Didactics Musical, Plastic and Corporal Expression, Faculty of Education Science, University of Granada, 18071 Granada, Spain; emelguizo@ugr.es (E.M.-I.); jlubago@ugr.es (J.L.U.-J.); josemalonsov@correo.ugr.es (J.M.A.-V.); 2 Faculty of Humanities and Social Sciences, Universidad Isabel I, 09003 Burgos, Spain

**Keywords:** health, social functioning, dietary adherence, emotional role, vitality

## Abstract

The Mediterranean diet is considered a healthy eating pattern. It has been shown to improve people’s quality of life. When a person suffers injuries, their quality of life suffers. This research aims to accomplish the following: (a) to study the differences in the effect of the health-related quality of life on injuries according to the degree of adherence to the Mediterranean diet, (b) to analyse the existing differences in the variables that make up the health-related quality of life according to the degree of adherence to the Mediterranean diet, and (c) to analyse the degree of adherence to the Mediterranean diet according to whether the participants have suffered any injury. The study was descriptive, cross-sectional, and exploratory in a sample of 556 physical education students. The PREDIMED questionnaire, the SF-36 questionnaire, and a self-administered questionnaire were used. The results showed that high adherence to the Mediterranean diet was associated with higher quality of life and lower injury rates. It was also observed that high adherence to the Mediterranean diet improved the effect of the quality of life on injuries. In conclusion, the Mediterranean diet is beneficial for the quality of life of young university students.

## 1. Introduction

There is now a large body of research that supports the benefits of physical activity [1,2]. These benefits are independent of the age, sex, place of residence, and socioeconomic status of the practitioner [3]. Moreover, the practice of physical activity is easily accessible to different people [3]. The training of people related to the physical-sport field is vital [4]. Prospective physical education teachers are those who are most exposed to sporting activities [4]. This places this population at a critical point for sport injuries [4]. Goosens et al. [4] showed that students involved in physical education perform more than a 1000 h of physical activity. This increases the risk of injury in this population [4].

In order for physical sport practice to not be harmful, some basic criteria must be met [5]. The first of these is suitability [6]. This states that the physical activity proposed must be adapted to the physical conditions of the individual [6]. The second principle is continuity [6]. This states that physical activity should be carried out on a regular basis [6]. The third principle is that of globalisation [6]. This states that the practice of physical-sport activities produces improvements in the other dimensions of the individual [6]. The last principle is that of progression [6]. This states that physical exercise should begin at a low intensity and for a short duration [6]. Failure to comply with these recommendations can lead to impact injuries that temporarily make it impossible to carry out physical-sport activities [6].

Suffering from injuries causes the quality of life of young people to be affected [7]. The quality of life can be related as subjective [8]. It depends on the context in which the person is and how perceives it [9]. Quality of life is defined as an individual perception of the position of life in the socio-cultural context, and in relation to personal goals, expectations, norms, and interests [9]. The quality-of-life concept is based on a definition that comprises a sense of well-being and happiness [10]. It excludes the health problems or illnesses that a person may suffer from [10]. In response to this, the concept of health-related quality of life was proposed. Health-related quality of life is part of a multidimensional approach [10]. It encompasses physical, mental, and related social symptoms, as well as limitations caused by illness [10]. Elements have been found to positively affect the quality of life and health-related quality of life. These include physical activity and diet [10].

The university education stage coincides with the time when students leave their home environment and start living independently [11]. This causes young people to begin to have greater control over their diet [11]. The eating habits of the home environment are abandoned, and new dietary patterns are acquired [11]. It has been observed that the large supply of ready meals, convenience, or lack of time together with inexperience in shopping are the main reasons why the dietary pattern followed varies [12]. The Mediterranean diet is one of the healthiest dietary patterns currently available [13]. It is mainly based on the moderate consumption of foods originating from the Mediterranean area [13]. It includes a moderate intake of unsaturated fats, fish, lean meats, fruits, vegetables, nuts, legumes, and a low intake of red meat and saturated fats [13]. The Mediterranean diet ensures adequate calorie and nutrient intake in sufficient quantities and proportions [13]. In addition, it contributes to the prevention of cardiovascular diseases, diabetes, cancer, degenerative diseases and, in general, to a longer life expectancy [13]. All these benefits contribute to the university students’ quality of life [13].

In addition to improvements in the quality of life, the Mediterranean diet helps prevent injuries [14]. Polyphenols (abundant in fruits, vegetables, and olive oil) and omega-3 fatty acids (abundant in fish) have been reported to protect musculoskeletal health [14]. It has been established that both low bone mass and low skeletal muscle mass increase susceptibility to low-impact fragility fractures [14]. The following research questions are proposed:

• Are there differences in the effect of health-related quality of life on injuries according to the degree of adherence to the Mediterranean diet? 

• Are there differences in health-related quality of life according to the degree of adherence to the Mediterranean diet?

• Are there differences in adherence to the Mediterranean diet in injured versus non-injured people?

The aims of the study related to the research hypotheses are shown below: 

O.1. To adjust and develop a multi-group education model according to the degree of adherence to the Mediterranean diet. 

O.2. To study the differences in the effect of the health-related quality of life on injuries according to the degree of adherence to the Mediterranean diet. 

• H.1. Participants showing high adherence to the Mediterranean diet will have a negative health-related quality of life effect on injuries.

• H.2. Participants who show high adherence to the Mediterranean diet will have higher scores on the variables that make up the health-related quality of life. 

O.3. To analyse the existing differences in the variables that make up the health-related quality of life according to the degree of adherence to the Mediterranean diet. 

• H.3. Participants who are injury-free will show a higher degree of adherence to the Mediterranean diet.

O.4. To analyse the degree of adherence to the Mediterranean diet according to whether the participants have suffered any injury.

• H.4. Participants who are uninjured will show greater adherence to the Mediterranean diet than those who are injured.

## 2. Materials and Methods

### 2.1. Design and Participants

This study is exploratory and comparative in nature. It presents a cross-sectional design. The sample is made up of 556 trainee physical education teachers from different grades of primary education in Andalusia. The age range was between 22 and 30 years (25.95 ± 2.768). All study subjects participated voluntarily after giving their informed consent. With respect to the sample reached, the sampling error was less than 3% for a confidence level of 95.0%.

### 2.2. Instruments and Variables

Various instruments were used to collect the data. The following is a list of the instruments that were used. 

**Own ad hoc questionnaire:** This was used to collect the variables of gender (male/female) and age of the participants. In addition, it was used to collect the injuries of the participants. The categorisation of this variable was divided into four areas where injuries could have occurred, and they are as follows: arms (None, Numbness, Overload, contracture), legs (None, Calf, Hamstring, quadriceps, Overload), lower back (None, lower back pain, Overload, contracture), and cervical (None, Bursitis over Apophysis, cervical algia and Open neck burn). The recommendations followed by Gómez-Montón et al. [15] and Poyatos et al. [16] were used to develop this categorisation.

**PREDIMED questionnaire:** It was used to measure the degree of adherence to the Mediterranean diet. The original version was elaborated and developed by Schröder et al. [17]. Due to the typology and characteristics of the sample, the version by Álvarez-Álvarez et al. [18] has been used. This questionnaire is made up of a total of 14 questions [18]. Once answered and depending on the final score, the questionnaire offers three levels of categorisation: low adherence, medium adherence, and high adherence. Cronbach’s alpha showed a value of α = 0.829. 

**SF-36 questionnaire:** To measure the health-related quality of life, the version adapted to Spanish by Alonso et al. [19] has been used. The instrument consists of 26 items rated on a Likert scale (1 = always; 5 = never). In addition, 10 items are rated on a 3-option Likert scale (1 = yes, it limits me a lot; 3 = it does not limit me at all). This makes a total of 36 items in the instrument. Finally, the health-related quality of life is measured through eight variables which are as follows: physical functioning (FF), physical role (RF), bodily pain (DC), general health (SG), vitality (VT), social functioning (FS), emotional role (RE), and mental health (SM). Cronbach’s alpha obtained a value of α = 0.798. 

### 2.3. Procedure

Before starting the research, a sweep of questionnaires was carried out to check which were the most reliable. A Google Form questionnaire was then created to house the instruments. After this, the different departments were contacted and invited to collaborate in this study. Most of them agreed to share the questionnaire. Only two negative responses were obtained. The participants gave their written informed consent to participate in this study. In addition, all participants were assured that after signing the informed consent, their data would be treated anonymously and confidentially. 

This study followed all the ethical aspects set out in the Declaration of Helsinki. In addition, to ensure greater ethical rigour, this research has been supervised and approved by an ethics committee (2966/CEIH/2022).

### 2.4. Data Analysis

IBM SPSS Statistics 25.0 software (IBM Corp., Armonk, NY, USA) was used for the relational analysis of the data. Before starting the data analysis, the distribution of the sample was studied. For this purpose, the Kolmogorov–Smirnov test was used. A significance value of less than 0.05 was obtained, so non-parametric tests were used to test the hypothesis. The Kruskal–Wallis test was used to compare more than two groups. When statistically significant differences (*p* < 0.05) were found, the Bonferroni test was used as a post hoc test to indicate differences between the groups. Cohen’s standardised *d* [20] was used to calculate statistical power. The value obtained can be classified into four levels: null (≤0.19), small (0.20–0.49), medium (0.50–0.79), and large (≥0.80).

**Figure 1:**
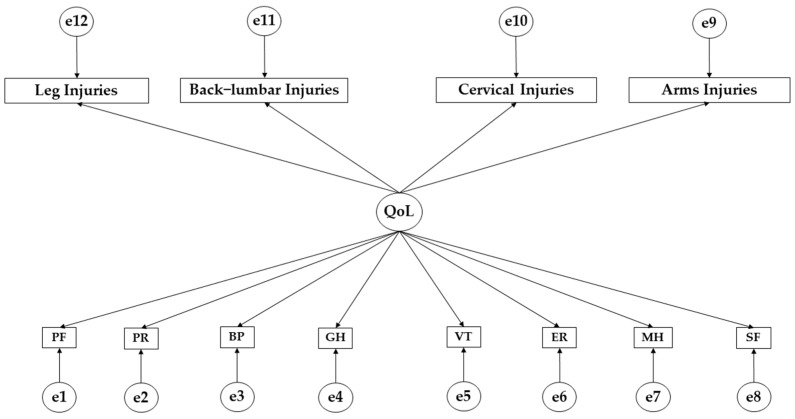
Theoretical model. **Note:** Health-related quality of life (QoL); physical function (PF); physical role (PR); bodily pain (BP); general health (GH); vitality (VT); Emotional Role (ER); mental health (MH); social functioning (SF).

IBM SPSS Amos 26.0 (IBM Corp., Armonk, NY, USA) was used for structural equation modelling. This analysis was used to test the fit of the proposed theoretical model to the data obtained through its suitability for testing the hypotheses [21]. Initially, a theoretical model was developed to establish the direction of the effects between the variables (Figure 1). The proposed model consists of endogenous and exogenous variables. The exogenous variables are those that have an effect on other variables [21]. The theoretical model is formed by an exogenous variable (QoL). Endogenous variables are those that receive the effect of the endogenous variables [21]. The model consists of twelve endogenous variables. Due to the characteristics of the endogenous variables, causal explanations could be added. These are based on the reliability of the measures and the fit indices. The inclusion of causal explanations has allowed the inclusion of measurement process errors in the endogenous variables. Regarding the direction of the effects, this has a unidirectional character in such a way that the effect only occurs in one direction. With regard to the study of statistical differences, the level of significance was set at *p* < 0.05.

The model fit was assessed through convergent validity and reliability or internal consistency. For this purpose, the mean variance extracted and the composite reliability were analysed. The values obtained for the mean variance extracted and composite reliability were adequate (VME = 0.60 and FC = 0.75). Also, Harman’s test was used to evaluate the common method variance problem [22] The goodness of fit of the theoretical model and the multigroup model was evaluated through the following indices: (a) X^2^, degrees of freedom (df), and *p*-values; (b) comparative fit index (CFI); (c) normalised fit index (NFI) analysis; (d) incremental fit index (IFI); (e) Tucker–Lewis index (TLI); (f) root mean square error of approximation (RMSEA). Following Hair et al. [23], an adequate model fit is obtained when X^2^ *p*-value ≥ 0.05, CFI > 0.90, and RMSEA ≤ 0.07.

With respect to the values obtained in the theoretical model, the values obtained are given below. X^2^ = 3.160; df = 16; *p* = 0.079; NFI = 0.935; IFI = 0.974; TLI = 0.988; CFI = 0.981; RMSEA = 0.039).

## 3. Results

Table 1 presents the descriptive analysis of the variables that make up the health-related quality of life according to the degree of adherence to the Mediterranean diet. Statistically significant differences (*p* < 0.05) were observed for physical function and vitality. For physical function, the participants with high adherence (7.80 ± 0.04) show higher recognition than those with medium adherence (7.62 ± 1.05) or low adherence (7.57 ± 0.63). For vitality, the students with high adherence (14.63 ± 1.93) show greater recognition than those with medium adherence (13.67 ± 2.03) or low adherence (12.89 ± 1.98). 

For general health, the participants with high adherence (15.37 ± 1.65) show higher recognition than those with medium adherence (14.85 ± 1.79) or low adherence (14.83 ± 1.82). For social functionality, greater recognition is shown for the participants with high adherence (7.18 ± 0.71). It is also observed that a high adherence to the Mediterranean diet denotes a higher level of mental health (21.25 ± 2.15). For bodily pain, the participants with low adherence have a higher recognition of this variable (4.08 ± 1.90). The young people with low adherence have a higher emotional role (5.82 ± 0.66). Finally, the participants with a high adherence to the Mediterranean diet have a higher emotional role (5.82 ± 0.88). 

Table 2 shows the injured areas with the type of injury according to the degree of adherence to the Mediterranean diet. For the arm area, differences (*p* < 0.05) were observed between the participants who had not suffered any injury (0.8014 ± 0.081) and those who had suffered contractures (0.7976 ± 0.082). For the lumbar–dorsal area, statistically significant differences are observed between those who have not suffered any injury (0.800 ± 0.082) and those who have suffered contractures (0.792 ± 0.900). For the legs, it was observed that the participants who had suffered quadricep injury had a greater adherence to the Mediterranean diet (0.8106 ± 0.084). Finally, for the cervical area, the participants who have presented cervical algia show a greater adherence to the Mediterranean diet (0.817 ± 0.072). 

**Figure 2:**
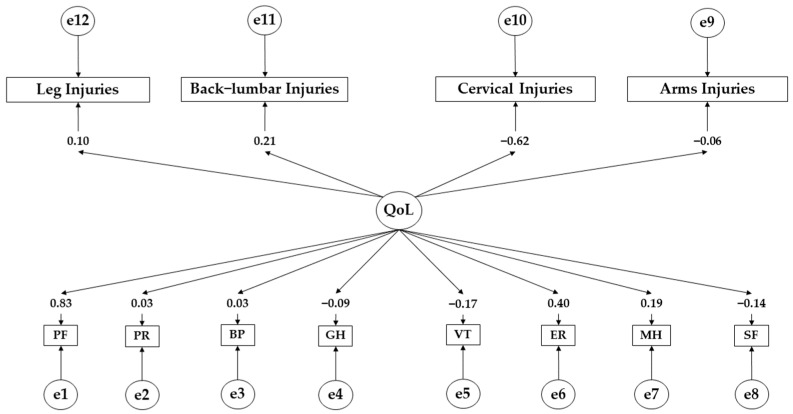
Theoretical model with regression weights for low adherers. **Note:** Quality of life (QoL); physical function (PF); physical role (PR); bodily pain (BP); general health (GH); vitality (VT); emotional role (ER); mental health (MH); social functioning (SF).

Table 3, Table 4 and Table 5 present the results of the structural equation models. Table 3 and Figure 2 show the standardised regression weights for the participants showing low adherence. For these participants, a positive effect of the health-related quality of life (QoL) on physical function (β = 0.825), physical role (β = 0.030), emotional role (β = 0.399; *p* < 0.05), bodily pain (β = 0.003), and mental health (β = 0.191) is observed. In contrast, a negative effect of the health-related quality of life (QoL) on vitality (β = −0.168), social functioning (β = −0.135), and general health (GH) (β = −0.086) was observed. Regarding injuries, a positive effect of the health-related quality of life (QoL) on leg injuries (β = 0.105) and back–lumbar injuries (β = 0.212) is denoted. In contrast, the health-related quality of life (QoL) has a negative effect on cervical injuries (β = −0.621; *p* < 0.05) and arm injuries (β = −0.057).

**Figure 3:**
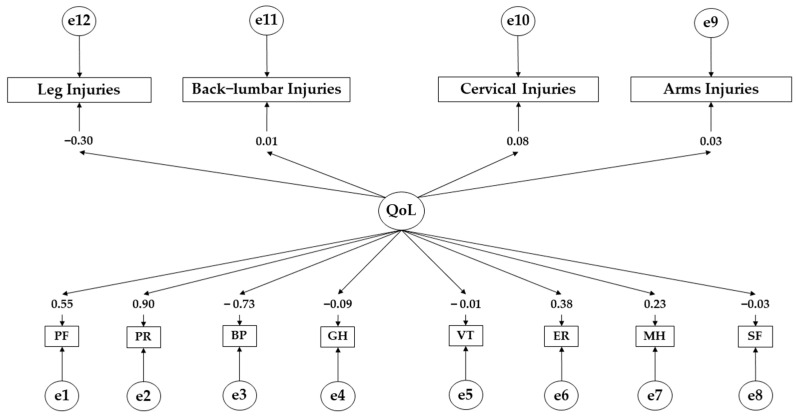
Theoretical model with regression weights for those with medium adherence. **Note:** Quality of life (QoL); physical function (PF); physical role (PR); bodily pain (BP); general health (GH); vitality (VT); emotional role (ER); mental health (MH); social functioning (SF).

Table 4 and Figure 3 present the standardised regression weights for young people showing medium adherence. A positive effect of the health-related quality of life on physical function (β = 0.551), physical role (β = 0.550; *p* < 0.001), emotional role (β = 0.900; *p* < 0.001), bodily pain (β = 0.386; *p* < 0.001), and vitality (β = 0.227; *p* < 0.05) is observed. Conversely, a negative effect of the health-related quality of life (QoL) on social functioning (β = −0.097) and general health (β = −0.091; *p* < 0.05) is obtained. Continuing with the effect of the health-related quality of life (QoL) on injury areas, a negative effect on leg injuries (β = −0.030) is obtained. In contrast, a positive effect on back–lumbar injuries (β = 0.004), cervical injuries (β = 0.083), and arm injuries (β = 0.026) is observed.

**Figure 4:**
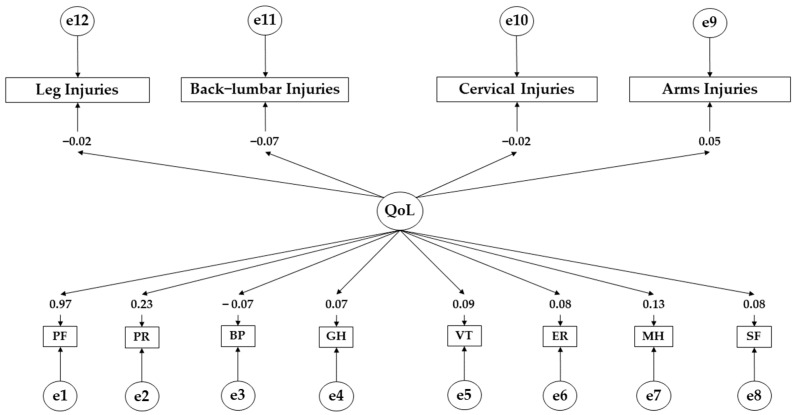
Theoretical model with regression weights for high adherents. **Note:** Quality of life (QoL); physical function (PF); physical role (PR); bodily pain (BP); general health (GH); vitality (VT); emotional role (ER); mental health (MH); social functioning (SF).

Table 5 and Figure 4 present the results obtained for participants with high adherence to the Mediterranean diet. A positive effect of the health-related quality of life on physical function (β = 0.969), physical role (β = 0.551), emotional role (β = 0.082), mental health (β = 0.128), vitality (β = 0.094), social functioning (β = 0.081), and general health (β = 0.071) is denoted. In contrast, a negative effect of the health-related quality of life (QoL) on bodily pain (β = −0.069) was observed. The effect of the health-related quality of life (QoL) on leg injuries (β = −0.016; *p* < 0.001), back–lumbar injuries (β = −0.066; *p* < 0.001), cervical injuries (β = −0.021; *p* < 0.001), and arm injuries (β = −0.047; *p* < 0.001) was also observed.

## 4. Discussion

Once the research questions, objectives, and research hypotheses have been answered, the results obtained are compared with those of another similar research.

The study shows that a high adherence to the Mediterranean diet has a positive impact on different areas of the health-related quality of life. Higher adherence to the Mediterranean diet reported higher scores on general health, social functioning, vitality, mental health, physical role, and physical function. The Mediterranean diet has reported benefits in the different areas that make up the human being [24]. High adherence to the Mediterranean diet is reported to reduce cardiovascular disease by 40% [25]. The Mediterranean diet benefits have also been found to reduce negative emotional states [26]. In the physical area, it has been reported that young people who show a high adherence to this dietary pattern show better physical fitness outcomes [27]. High adherence to the Mediterranean diet has shown improvements in musculoskeletal fitness and overall physical fitness [26]. All the above highlight the health benefits of positive adherence to the Mediterranean diet. This analysis indicates that the young people with a low adherence show higher emotional role and bodily pain. It has been shown that in populations with osteoarthritis, the Mediterranean diet helps to reduce bodily pain [28]. Furthermore, a reduction in chronic pain has been observed with a high adherence to the Mediterranean diet [29]. For the emotional role, it is noted that the young people with a high adherence show worse results. It has been observed that a process of emotional overeating is taking place in the adolescent and adult population [30]. This consists of eating unhealthy foods to reduce negative emotional states [31].

The study related to injuries and Mediterranean adherence has reported that for some body segments, participants who have not had any injuries have shown a higher adherence to the Mediterranean diet. High adherence to the Mediterranean diet has reported benefits at the bone and muscle level [32]. This strengthening of the musculoskeletal system helps to prevent possible injuries sustained during sport [33]. At a competitive level, it has been reported that the Mediterranean diet provides the intake of macronutrients and micronutrients to boost muscle contraction [34]. The diet followed has been shown to play an important role in preventing injury [35]. There are other elements to consider in injury prevention such as internal factors and external factors [35]. Internal factors refer to factors within the body such as age, gender, and fitness [35]. External factors refer to training planning, adequate warm-up, previous fatigue, and overtraining [35].

The results obtained in the multi-group model reveal that there are differences between the health-related quality of life and injury site. The effect of the quality of life on leg injuries shows a positive effect for the young people with low adherence. A negative effect is denoted for the young people with medium and high adherence. Nutrition together with the rehabilitation process helps to improve the mental and physical function of the person [36]. Post-injury is the best time to improve eating attitudes and behaviours [37]. This process of change leads to an improvement in people’s health-related quality of life [35]. This leads to the healing process and/or improves the subsequent performance of the affected area [34]. It has been shown that a high adherence to the Mediterranean diet was associated with an increase in muscle mass and explosive leg power [38]. This is positively associated with increased lower body strength, which reduces the risk of leg injuries [39].

The effect of the health-related quality of life on lower back and hip injuries is positive for the young people who show low or medium adherence to the Mediterranean diet. In contrast, a negative effect of the health-related quality of life on injuries in this area is denoted. High adherence to a high-protein diet has been found to be inversely associated with the prevalence of chronic lower back pain [40]. The beneficial effects of the Mediterranean diet on cardiovascular disease, diabetes, obesity, and bone fragility have been widely recognised [41]. These conditions are, in turn, associated with an increased risk of injury [42]. For back injuries, it has been observed that asthma, diabetes, and osteoarthritis were associated with an increased number of lower back injuries [43].

The effect of the health-related quality of life on neck injuries shows a positive effect for medium adherence. Negative effects between these variables are denoted for the participants with low and high adherence to the Mediterranean diet. This effect is larger for the participants with low adherence. It has been found that the Mediterranean diet by promoting the intake of bioactive antioxidant and anti-inflammatory components may have a protective effect on muscular and skeletal health [7]. In order to strengthen muscles in a particular area, this must be combined with training [38].

The effect of the health-related quality of life shows a greater negative effect for the young people with low adherence to the Mediterranean diet. Different results were found by Julián et al. [44]. In their study, it was found that the Mediterranean diet helps muscle strengthening [44]. This helps to prevent possible injuries sustained during physical activity [44].

This research has responded to the objectives, hypotheses, and research questions. The study has several limitations. This research is cross-sectional. This reflects the fact that data were only collected at a single point in time. In addition, due to the type of study, other variables that directly influence the research variables have been left out. The instruments used are another limitation. Instruments have been used that have shown a high degree of reliability. Despite this, these instruments have an intrinsic error related to the measurement process. Another limitation is the decline in this diet due to local or traditional culinary habits in the area where the data were collected. The last limitation relates to the fact that the study lacks objective observations such as the participants’ body synthesis (fat mass, lean mass) and clinical measurements.

As a future study, an intervention programme could be carried out with a control group and an experimental group. In this, the control group could be assigned the intake of healthy foods that help to strengthen muscles and bones in physical education students. In addition, most of the research on this subject has been carried out on elite athletes. It would be interesting to know the state of the art in university students from various branches of university study.

## 5. Conclusions

The main conclusions found in this research highlight that a high adherence to the Mediterranean diet brings greater benefits in the different areas that make up the health-related quality of life. It is noted that with respect to the areas of injury, there are differences in the type of injury and adherence to the Mediterranean diet. It is concluded that there are differences according to the degree of adherence to the health-related quality of life in relation to injuries. The participants with a high degree of adherence to the Mediterranean diet were found to have a negative effect of the health-related quality of life on injuries. As a general assessment, it is observed that a high adherence to the Mediterranean diet brings health benefits to individuals.

Derived from these conclusions, the need arises to seek their applicability. The importance of positive adherence to the Mediterranean diet should be conveyed in the educational sphere. Through the discipline of physical education, habits that have a positive impact on the quality of life should be encouraged, as this prevents injuries and illnesses.

## Figures and Tables

**Table 1:** Descriptive analysis of the health-related quality of life as a function of adherence to the Mediterranean diet.

		M	SD	F	*p*	ES (*d*)	95% Confidence Interval
General Health	Low Adherence	14.83	1.82	1.944	0.144	NP	NP
	Medium Adherence	14.85	1.79				
	High Adherence	15.37	1.65				
Social Functioning	Low Adherence	6.90	0.54	1.630	0.196	NP	NP
	Medium Adherence	6.91	0.92				
	High Adherence	7.18	0.71				
Vitality	Low Adherence	12.89	1.98	4.246	0.015	0.493 ^a^	[0.205–0.781] ^a^
	Medium Adherence	13.67	2.03				
	High Adherence	14.63	1.93				
Mental Health	Low Adherence	19.67	1.84	0.269	0.764	NP	NP
	Medium Adherence	19.51	1.88				
	High Adherence	21.25	2.15				
Bodily Pain	Low Adherence	4.08	1.90	0.422	0.656	NP	NP
	Medium Adherence	3.93	1.97				
	High Adherence	3.85	2.24				
Emotional Role	Low Adherence	5.82	0.66	2.207	0.111	NP	NP
	Medium Adherence	5.58	0.91				
	High Adherence	5.75	0.70				
Physical Role	Low Adherence	5.75	1.13	0.640	0.527	NP	NP
	Medium Adherence	5.58	1.03				
	High Adherence	5.82	0.88				
Physical Function	Low Adherence	7.57	0.63	3.251	0.039	0.048 ^a^	[0.239; 0.335] ^a^
	Medium Adherence	7.62	1.05				
	High Adherence	7.80	0.04				

**Note:** ^a^ Differences between low adherence and medium adherence. Mean (M); standard deviation (SD); Fisher’s test (F); effect size (ES).

**Table 2:** Zones with the types of lesions according to the degree of adherence to the Mediterranean diet.

		M	SD	F	*p*	ES (*d*)	95% Confidence Interval
Arms	None	0.801	0.081	0.444	0.022	0.215 ^a^	[0.193–0.642] ^a^
	Numbness	0.794	0.084				
	Overload	0.785	0.002				
	Contracture	0.797	0.082				
Legs	None	0.799	0.083	0.508	0.730	NP	NP
	Calf	0.795	0.081				
	Hamstring	0.802	0.068				
	Quadriceps	0.810	0.084				
	Overload	0.797	0.081				
Back–lumbar	None	0.800	0.082	0.212	0.048	0.586 ^a^	[0.345–0.708] ^a^
	Lower back pain	0.801	0.077				
	Overload	0.798	0.065				
	Contracture	0.792	0.900				
Cervical	None	0.798	0.084	2.494	0.059	NP	NP
	Cervical algia	0.817	0.072				
	Contracture	0.805	0.075				

**Note:** ^a^ difference between no injury and contracture; mean (M); standard deviation (SD); Fisher’s test (F); effect size (ES).

**Table 3:** Standardised regression weights for those with low adherence.

Associations between Variables	R.W.				S.R.W.
	Estimates	S.E.	C.R.	*p*	Estimates
PF ← QoL	1.000				0.825
PR ← QoL	0.064	0.357	0.181	0.857	0.030
ER← QoL	0.508	0.244	2.081	**	0.399
BP ← QoL	0.009	0.597	0.016	0.988	0.003
MH ← QoL	0.668	0.596	1.121	0.262	0.191
VT ← QoL	−0.638	0.643	−0.993	0.321	−0.168
SF ← QoL	−0.142	0.176	−0.806	0.420	−0.135
GH ← QoL	−0.272	0.523	−0.520	0.603	−0.086
Leg Injuries ← QoL	0.209	0.330	0.633	0.526	0.105
Back–Lumbar Injuries ← QoL	0.322	0.261	1.233	0.218	0.212
Cervical Injuries ← QoL	−1.286	0.512	−2.514	**	−0.621
Arm Injuries ← QoL	−0.047	0.137	−0.347	0.729	−0.057

**Note:** Quality of life (QoL); physical function (PF); physical role (PR); bodily pain (BP); general health (GH); vitality (VT); emotional role (ER); mental health (MH); social functioning (SF). ** *p* ≤ 0.05.

**Table 4:** Standardised regression weights for those with average adherence.

Associations between Variables	R.W.				S.R.W.
	Estimates	S.E.	C.R.	*p*	Estimates
PF ← QoL	1.000				0.551
PR ← QoL	1.601	0.103	15.497	***	0.550
ER← QoL	0.605	0.060	10.093	***	0.900
BP ← QoL	−2.456	0.156	−15.750	***	−0.386
MH ← QoL	0.735	0.117	6.308	***	−0.726
VT ← QoL	−0.340	0.122	−2.781	0.005	0.227
SF ← QoL	−0.054	0.055	−0.980	0.327	−0.097
GH ← QoL	−0.281	0.108	−2.611	0.009	−0.091
Leg Injuries ← QoL	−0.055	0.065	−0.850	0.395	−0.030
Back–Lumbar Injuries ← QoL	0.005	0.051	0.105	0.916	0.004
Cervical Injuries ← QoL	0.170	0.072	2.368	0.018	0.083
Arm Injuries ← QoL	0.021	0.029	0.737	0.461	0.026

**Note:** Quality of life (QoL); physical function (PF); physical role (PR); bodily pain (BP); general health (GH); vitality (VT); emotional role (ER); mental health (MH); social functioning (SF). Regression weights (R.W.); standardised regression weights (S.R.W.); Estimation error (S.E.); Critical Ratio (C.R.). *** *p* ≤ 0.001.

**Table 5:** Standardised regression weights for those with high adherence.

Associations between Variables	R.W.				S.R.W.
	Estimates	S.E.	C.R.	*p*	Estimates
PF ← QoL	1.000				0.969
RP ← QoL	8.041	18.822	0.427	0.669	0.226
R← QoL	2.385	5.877	0.406	0.685	0.082
BP ← QoL	5.977	14.789	0.404	0.686	−0.069
MH ← QoL	13.393	32.568	0.411	0.681	0.128
VT ← QoL	8.325	20.443	0.407	0.684	0.094
SF ← QoL	2.286	5.635	0.406	0.685	0.081
GH ← QoL	6.292	15.559	0.404	0.686	0.071
Leg Injuries ← QoL	0.708	1.950	−0.363	***	−0.016
Back–Lumbar Injuries ← QoL	−1.950	4.829	−0.404	***	−0.066
Cervical Injuries ← QoL	−0.933	2.464	−0.379	***	−0.021
Arm Injuries ← QoL	0.761	1.904	−0.400	***	−0.047

**Note:** Quality of life (QoL); physical function (PF); physical role (PR); bodily pain (BP); general health (GH); vitality (VT); emotional role (ER); mental health (MH); social functioning (SF). Regression weights (R.W.); standardised regression weights (S.R.W.); Estimation error (S.E.); Critical Ratio (C.R.); *** *p* ≤ 0.001.

## Data Availability

The data used to support the findings of the current study are available from the corresponding author upon request.
